# RUNX1 is a promising prognostic biomarker and related to immune infiltrates of cancer-associated fibroblasts in human cancers

**DOI:** 10.1186/s12885-022-09632-y

**Published:** 2022-05-09

**Authors:** Zhouting Tuo, Ying Zhang, Xin Wang, Shuxin Dai, Kun Liu, Dian Xia, Jinyou Wang, Liangkuan Bi

**Affiliations:** Department of Urology, The Second Affiliated Hospital of Anhui Medical University, Hefei, China

**Keywords:** RUNX1, Prognostic biomarker, TCGA, Cancer-associated fibroblasts

## Abstract

**Background:**

Runt-related transcription factor 1 (RUNX1) is a vital regulator of mammalian expression. Despite multiple pieces of evidence indicating that dysregulation of RUNX1 is a common phenomenon in human cancers, there is no evidence from pan-cancer analysis.

**Methods:**

We comprehensively investigated the effect of RUNX1 expression on tumor prognosis across human malignancies by analyzing multiple cancer-related databases, including Gent2, Tumor Immune Estimation Resource (TIMER), Gene Expression Profiling Interactive Analysis (GEPIA), the Human Protein Atlas (HPA), UALCAN, PrognoScan, cBioPortal, STRING, and Metascape.

**Results:**

Bioinformatics data indicated that RUNX1 was overexpressed in most of these human malignancies and was significantly associated with the prognosis of patients with cancer. Immunohistochemical results showed that most cancer tissues were moderately positive for granular cytoplasm, and RUNX1 was expressed at a medium level in four types of tumors, including cervical cancer, colorectal cancer, glioma, and renal cancer. RUNX1 expression was positively correlated with infiltrating levels of cancer-associated fibroblasts (CAFs) in 33 different cancers. Moreover, RUNX1 expression may influence patient prognosis by activating oncogenic signaling pathways in human cancers.

**Conclusion:**

Our findings suggest that RUNX1 expression correlates with patient outcomes and immune infiltrate levels of CAFs in multiple tumors. Additionally, the increased level of RUNX1 was linked to the activation of oncogenic signaling pathways in human cancers, suggesting a potential role of RUNX1 among cancer therapeutic targets. These findings suggest that RUNX1 can function as a potential prognostic biomarker and reflect the levels of immune infiltrates of CAFs in human cancers.

**Supplementary Information:**

The online version contains supplementary material available at 10.1186/s12885-022-09632-y.

## Background

Despite great advances in the rapid diagnosis and treatment of tumors, cancer remains a major cause of death [1]. Given the increased morbidity and mortality among patients with cancer, it is necessary to further understand the pathogenesis of this disease to improve patient outcomes. Using the analysis of public data from the Cancer Genome Atlas (TCGA) project and the Gene Expression Omnibus (GEO) database [2, 3], we can now understand the function of certain genes in human cancer.

Runt-related transcription factors (RUNXs) are involved in the regulation of several biological processes in mammals. For example, RUNX family members RUNX1, 2, and 3 play important roles in skeletal development, while the transcription factor RUNX2 is required for osteoblast differentiation and chondrocyte maturation [4]. RUNX family members bind to the same non-DNA-binding core binding factor-beta (CBF-β) subunit to form a heterodimer, but they exhibit distinct expression patterns [5]. RUNX1 is widely expressed in mammalian cells and is reported to be dysregulated in many human cancers [4, 6]. Overexpression of RUNX1 has also been observed in hepatocellular carcinoma [7] and gastric cancer [8]. Interestingly, RUNX1 promotes the development of ovarian and skin cancers [9, 10], but exhibits tumor-suppressive activity in lung and prostate cancers [11, 12]. *RUNX1* mutations are closely related to tumorigenesis in leukemia [13] and breast cancer [14]. Moreover, it has been reported that RUNX1 phosphorylation involves in osteolytic bone destruction in ERα-positive breast cancer [15]. However, whether RUNX1 is involved in the pathogenesis of multiple tumors through a common signaling pathway remains unclear.

In our study, we systematically explored the effect of RUNX1 expression on the prognosis associated with several human cancers. Our findings indicate that RUNX1 expression is increased in various tumors, and thus may be linked to tumor progression and patient prognosis. Moreover, RUNX1 expression levels can reflect the infiltration of cancer-associated fibroblasts (CAFs) in tumor tissues.

## Methods

### Data collection and processing

We first studied the expression levels of the RUNX1 in human cancer using the Gent2 database (http://gent2.appex.kr/gent2/), TIMER database (https://cistrome.shinyapps.io/timer/), UALCAN database (http://ualcan.path.uab.edu), Gene Expression Profiling Interactive Analysis (GEPIA) database (http://gepia.cancer-pku.cn/), and Human protein atlas (HPA, https://www.proteinatlas.org). Subsequently, we evaluated the prognostic role of RUNX1 in cancer patients by using the PrognoScan database (http://gibk21.bse.kyutech.ac.jp/PrognoScan/index.html) and the GEPIA database. Next, we selected the “TCGA Pan Cancer Atlas Studies” in the cBioPortal web (https://www.cbioportal.org/) for analysis of the genetic alteration characteristics of *RUNX1* in human cancer. The immunological role of RUNX1 was analyzed using the TIMER database. Finally, we analyzed the co-expression genes of RUNX1 in the STRING (https://string-db.org/) database, and the related functional predictions between RUNX1 and their co-expressed genes in the Kyoto encyclopedia of genes and genomes (KEGG, https://www.genome.jp/kegg/), gene ontology (GO, http://geneontology.org/) and Metascape (https://metascape.org/gp/index.html).

### Gent2 database analysis

The Gent2 database (http://gent2.appex.kr/gent2/), an online cancer microarray database, was used to analyze the transcriptional expression of *RUNX1* in different human cancers [16].

### Tumor Immune Estimation Resource (TIMER) database analysis

The TIMER database (https://cistrome.shinyapps.io/timer/) can be used to analyze the correlation between gene expression and the level of immune cell infiltration in various human cancers [17]. The “Diffexp module” was used in this study to evaluate the *RUNX1* expression across human cancers. Next, a correlation analysis was performed between *RUNX1* expression and infiltrating levels of CD8 + T cells and cancer-associated fibroblasts (CAFs) in different types of tumors both by “gene modules” and “outcome modules”.

### Human Protein Atlas (HPA) database analysis

The HPA project (https://www.proteinatlas.org) includes information on the distribution of more than 24,000 human proteins in tissues and cells [18]. Thus, we searched the HPA website to analyze RUNX1 protein expression in both human cancer and normal tissues. Immunostaining intensity and patient information corresponding to the different cancer types are available on this website.

### Gene Expression Profiling Interactive Analysis (GEPIA) database analysis

The GEPIA website (http://gepia2.cancer-pku.cn/) has extensive gene expression data from TCGA and the Genotype-Tissue Expression (GTEx) databases [19]. In this study, we analyzed RUNX1 expression in human cancers by "Expression on Box Plots" mode, and then used the "Expression on Box Plots" and "Survival Plots" to analyze the correlation between RUNX1 expression and tumor stage and prognosis across human cancers, including overall survival (OS) and disease-free survival (DFS).

### UALCAN database analysis

The UALCAN database (http://ualcan.path.uab.edu) provides publicly available data from TCGA [20]. In this study, TCGA analysis was conducted to investigate DNA methylation of the *RUNX1* promoter in different types of cancer.

### PrognoScan database survival analysis

The PrognoScan database (http://gibk21.bse.kyutech.ac.jp/PrognoScan/index.html) was used to analyze the relationship between RUNX1 expression and clinical outcomes [21]. In this study, we selected all cancer types and a *P*-value < 0.05 as the threshold.

### The cBioPortal database analysis

The cBioPortal (https://www.cbioportal.org) has been used to analyze the information on genetic alterations in various cancer genomic datasets [22]. In our study, we analyzed the alteration frequency of *RUNX1* in different cancers based on data from “TCGA Pan-Cancer Atlas Studies,” and summarized mutated site information of the *RUNX1* gene. We obtained the alteration frequency and the information on genetic mutations of the *RUNX1* gene across human cancers using the “Cancer Type Summary” and “Mutations” modes, respectively.

### STRING database analysis

The STRING database (https://string-db.org/cgi/input?sessionId=bL2ZI4D088fF) is used to model protein–protein interaction (PPI) networks [23]. In our study, the PPI network of the RUNX1 protein was visualized using the following filters: “full STRING network,” “evidence,” “experiments,” “low confidence (0.150),” and “no more than 50 interactors” in the 1st shell.

### Metascape database analysis

The Metascape database (https://metascape.org/gp/index.html) is a publicly available website for functional gene analysis [24]. Enriched analyses of *RUNX1* and its neighboring genes were performed using Metascape to investigate the possible functional mechanisms of RUNX1, including Gene Ontology (GO) and Kyoto Encyclopedia of Genes and Genomes (KEGG) pathway analyses [25]. These terms were considered significant, with a *P*-value < 0.01, count > 3, and enrichment factor > 1.5. A two-tailed *P* < 0.05 was considered statistically significant.

### Statistical analysis

Statistical analyses of all data were performed using the statistical software from all online databases. Statistical significance was set at *P* < 0.05.

## Results

### Transcriptional levels of *RUNX1* in the pan-cancer analysis

**Fig. 1:**
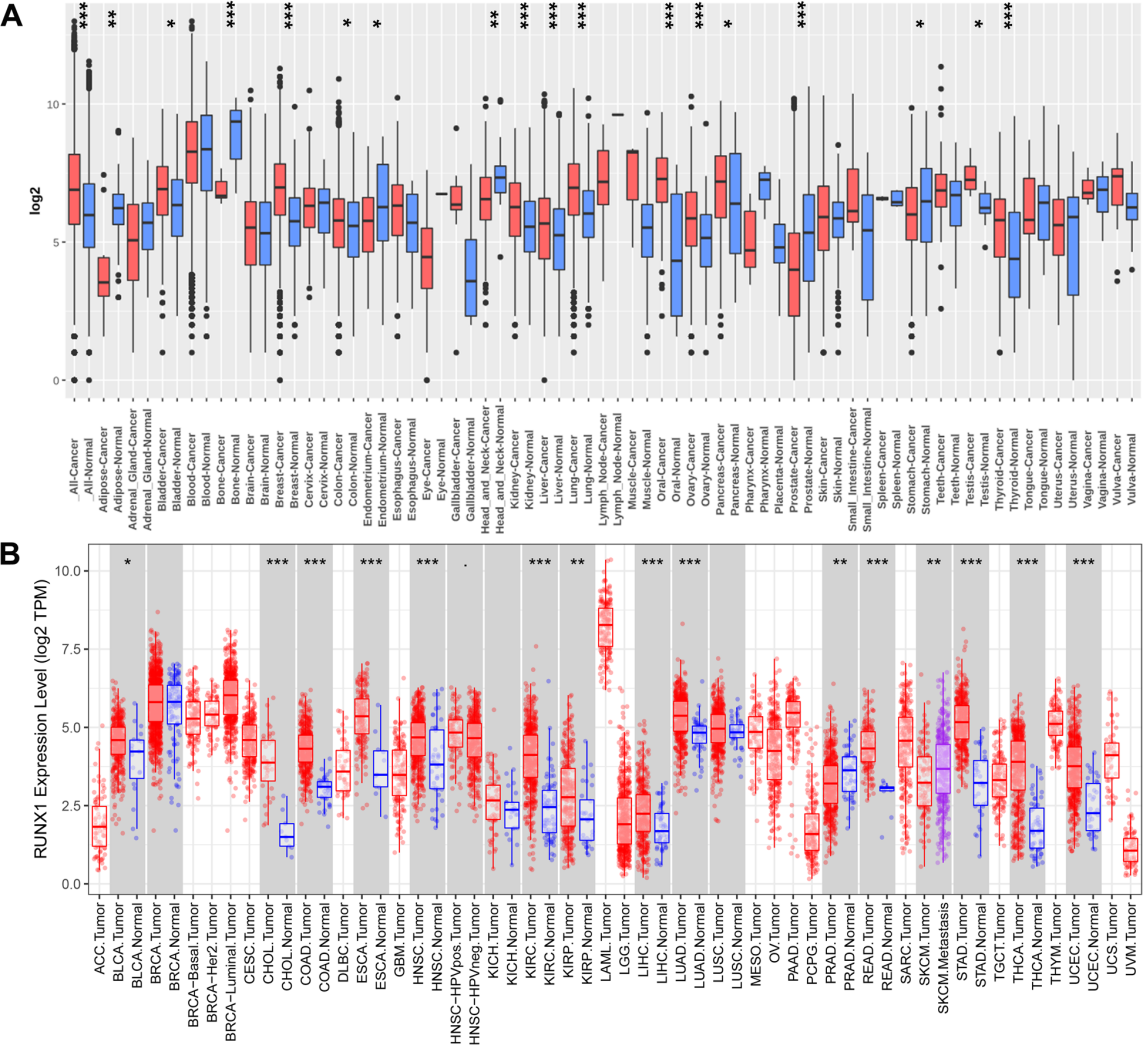
RUNX1 expression analysis in pan-cancer. **A**. Increased or decreased RUNX1 in datasets of different cancers compared with normal tissues in the Gent2 database; **B**. RUNX1 expression profile across all tumor samples and paired normal tissues determined by TIMER database. * *P* < 0.05; ** *P* < 0.01; *** *P* < 0.001

**Fig. 2:**
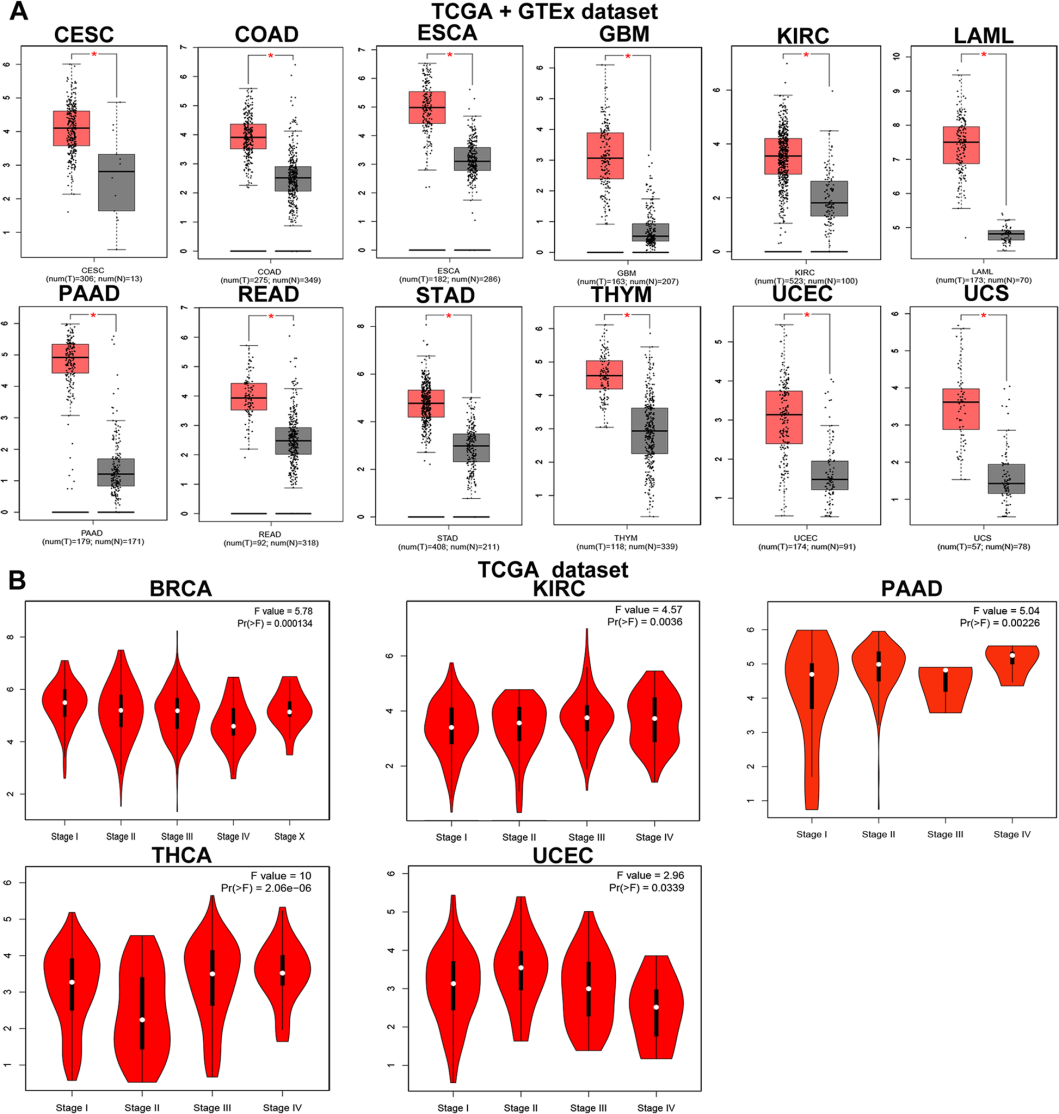
Correlation between RUNX1 expression and clinicopathological parameters in human cancers (GEPIA database). **A**. The expression of RUNX1 in different cancer tissues and normal tissues; **B**. Correlation between RUNX1 expression and tumor stage in different human cancers. *Indicate that the results are statistically significant

We first analyzed the mRNA expression levels of *RUNX1* across human cancers and paired normal samples by utilizing the HG-U133 microarray (GPL570 platform) of the Gent2 database (Fig. 1A). Compared with normal samples, *RUNX1* was upregulated in a variety of tumors, including bladder cancer, breast cancer, colorectal cancer, kidney cancer, liver cancer, lung cancer, oral cancer, ovary cancer, pancreatic cancer, testis cancer, and thyroid cancer (all *P* < 0.05). Meanwhile, *RUNX1* expression was decreased in multiple datasets, including adipose cancer, bone cancer, endometrium cancer, head and neck cancer, prostate cancer, and stomach cancer (all *P* < 0.05). Second, we verified the differences in *RUNX1* expression using the TIMER database. As shown in Fig. 1B, compared to normal samples, overexpression of *RUNX1* was observed in 17 pathological types of tumors, including bladder urothelial carcinoma (BLCA), cervical squamous cell carcinoma and endocervical adenocarcinoma (CESC), cholangiocarcinoma (CHOL), colon adenocarcinoma (COAD), esophageal carcinoma (ESCA), glioblastoma multiforme (GBM), head and neck squamous cell carcinoma (HNSC), kidney renal clear cell carcinoma (KIRC), kidney renal papillary cell carcinoma (KIRP), liver hepatocellular carcinoma (LIHC), lung adenocarcinoma (LUAD), rectum adenocarcinoma (READ), stomach adenocarcinoma (STAD), thyroid carcinoma (THCA), and uterine corpus endometrial carcinoma (UCEC); however, *RUNX1* expression was reduced in prostate adenocarcinoma (PRAD) (all *P* < 0.05). Third, we supplemented the normal tissue expression data from the GTEx dataset as a control group and retrieved the mRNA expression status of *RUNX1* in human tumors using the GEPIA website (Fig. S1). The results showed that *RUNX1* expression was higher in CESC, COAD, ESCA, GBM, KIRC, acute myeloid leukemia (AML), pancreatic adenocarcinoma (PAAD), READ, STAD, thymoma (THYM), UCEC, and uterine carcinosarcoma (UCS) than in adjacent normal tissue samples (all *P* < 0.05; Fig. 2A). However, no statistical differences were found for other tumors. Finally, we assessed the protein levels of RUNX1 in the tissues based on the results from the HPA database (Fig. S2A-B) and found that most of the cancer tissues were moderately positive for granular cytoplasm.

### Associations between the mRNA levels of RUNX1 and clinicopathological parameters across human cancers

**Fig. 3:**
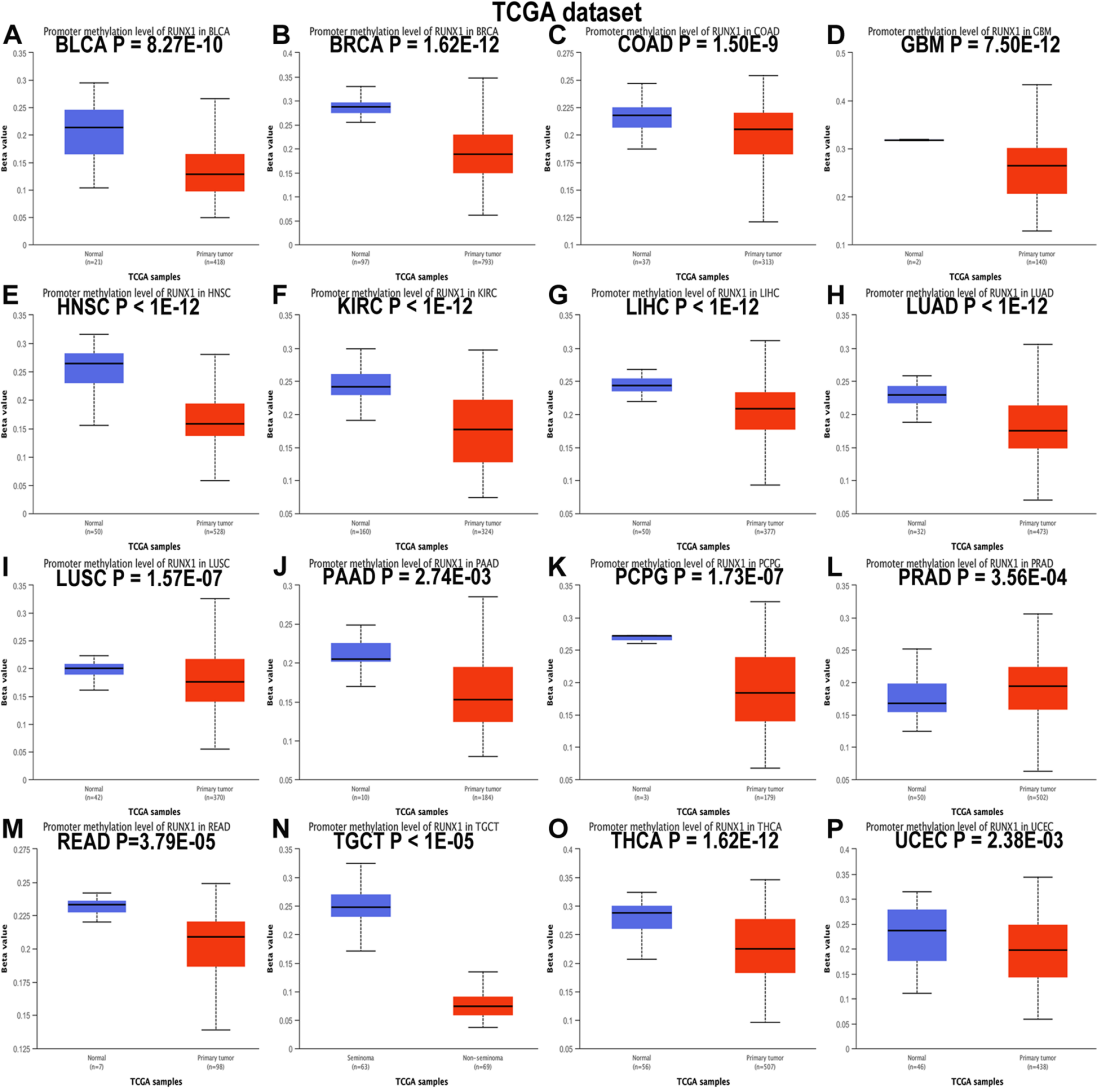
RUNX1 promoter methylation analysis in pan-cancer based on UALCAN database. **A**-**P**.The promoter methylation of RUNX1 in bladder urothelial carcinoma (BLCA), breast invasive carcinoma (BRCA), colon adenocarcinoma (COAD), glioblastoma multiforme (GBM), head and neck squamous cell carcinoma (HNSC), kidney renal clear cell carcinoma (KIRC), liver hepatocellular carcinoma (LIHC), lung adenocarcinoma (LUAD), lung squamous cell carcinoma (LUSC), pancreatic adenocarcinoma (PAAD), pheochromocytoma and paraganglioma (PCPG), prostate adenocarcinoma (PRAD), rectal adenocarcinoma (READ), testis germ cell tumor (TGCT), thyroid carcinoma (THCA), and uterine corpus endometrial carcinoma (UCEC)

We analyzed *RUNX1* expression in tumors at different stages using the GEPIA website (Fig. 2B). The expression levels of *RUNX1* varied significantly in Breast invasive carcinoma(BRCA), KIRC, PAAD, THCA, and UCEC (all *P* < 0.05). Next, we used the UALCAN database to explore the correlation between *RUNX1* expression levels and promoter methylation in human cancers. The results suggested that the promoter region of *RUNX1* exhibited hypomethylation in a variety of tumors, including BLCA, BRCA, COAD, GBM, HNSC, KIRC, LIHC, LUAD, lung squamous cell carcinoma (LUSC), PAAD, pheochromocytoma and paraganglioma (PCPG), READ, testicular germ cell tumors (TGCT), THCA, and UCEC, but hypermethylation in PRAD (Fig. 3A-P; all *P* < 0.05).

### RUNX1 prognosis analysis in different human cancers

**Fig. 4:**
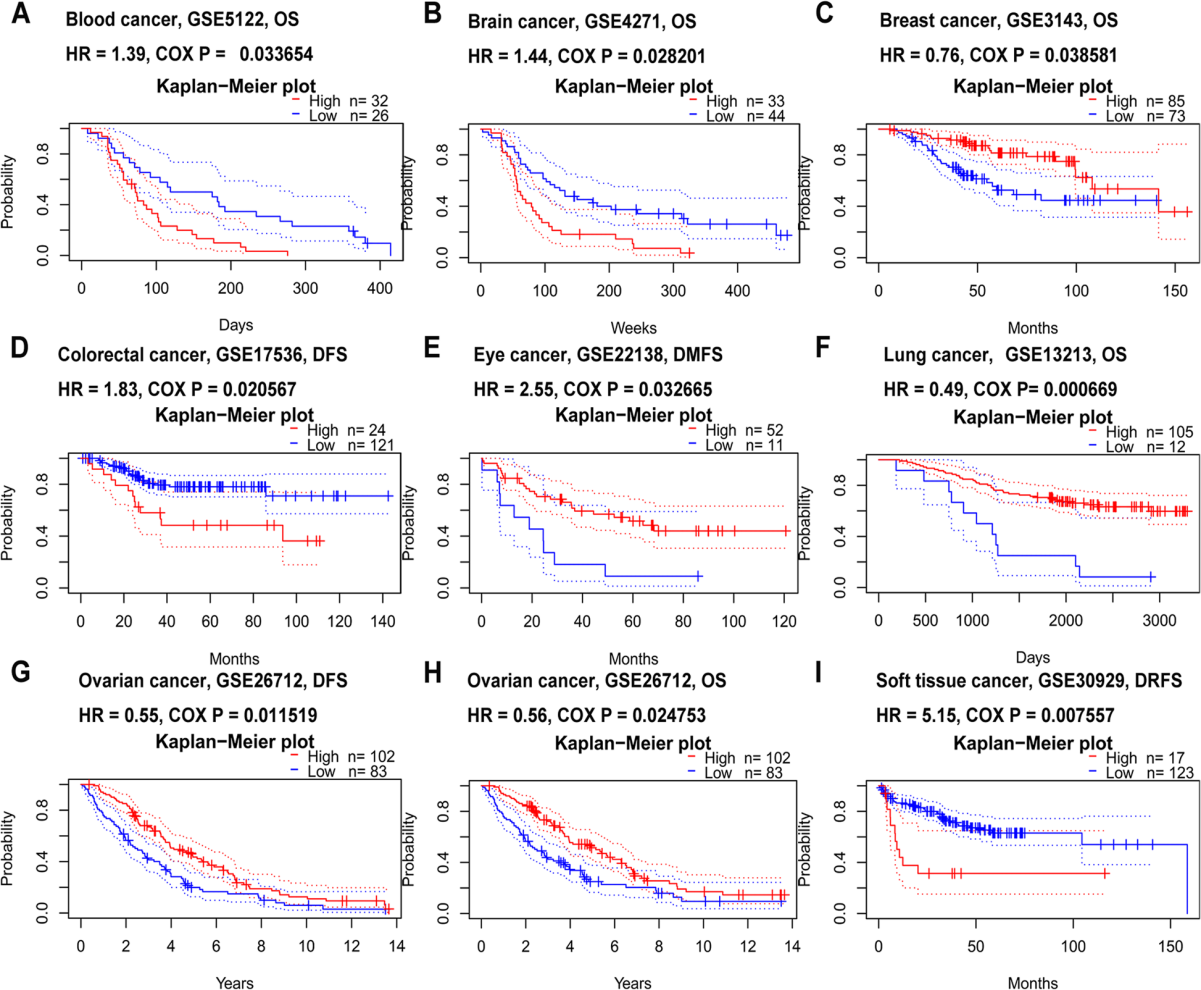
The prognostic values of RUNX1 in different human cancers by PrognoScan database. **A**. Overall survival (OS) curve of blood cancer; **B**. Overall survival (OS) curve of brain cancer; **C**. Overall survival (OS) curve of breast cancer; **D**. Disease free survival (DFS) curve of colorectal cancer; **E**. Distant metastasis free survival (DMFS) curve of eye cancer; **F**. Overall survival (OS) curve of lung cancer; **G**. and **H**. Disease free survival (DFS) and overall survival (OS) curve of ovarian cancer; **I**. Distant recurrence free survival (DRFS) curve of soft tissue cancer

**Fig. 5:**
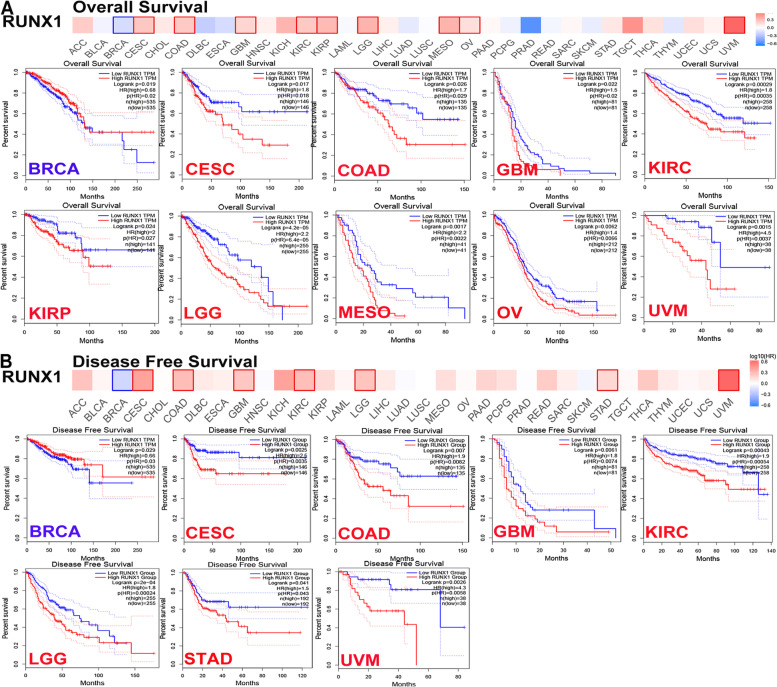
The prognostic value of RUNX1 in different human cancers by GEPIA database. **A**. Overall survival analysis of RUNX1 expression in different tumors; **B**. Disease-free survival analysis of RUNX1 expression in different tumors

To assess the prognostic role of RUNX1 in patients with cancer, we conducted a prognosis analysis across human cancers using PrognoScan and GEPIA. First, we observed a correlation between RUNX1 expression and prognosis in 8 of the 13 types of cancer using the PrognoScan database (Table S1; Fig. 4A-I). Our results suggest that RUNX1 expression plays a detrimental role in four cancer types, including blood, brain, colorectal, and soft tissue cancers. However, they also suggest that RUNX1 plays a protective role in four other cancers, including breast, eye, lung, and ovarian cancers. Second, we studied the role of RUNX1 in human cancers using GEPIA (Table S2; Fig. 5A-B). Notably, RUNX1 had a negative overall effect on cancer (OS: total log-rank *P* = 0, HR = 1.4; DFS: total log-rank *P* = 0.29, HR = 0.96). High RUNX1 expression levels were linked to worse OS and DFS in CESC, COAD, GBM, KIRC, brain lower-grade glioma (LGG), and uveal melanoma (UVM), but were related to better OS and DFS in BRCA. Meanwhile, mesothelioma (MESO), ovarian cancer (OV), and STAD outcomes were found to have a negative correlation with RUNX1 expression. However, RUNX1 expression has no significant effect on the prognosis of other cancers.

**Fig. 6:**
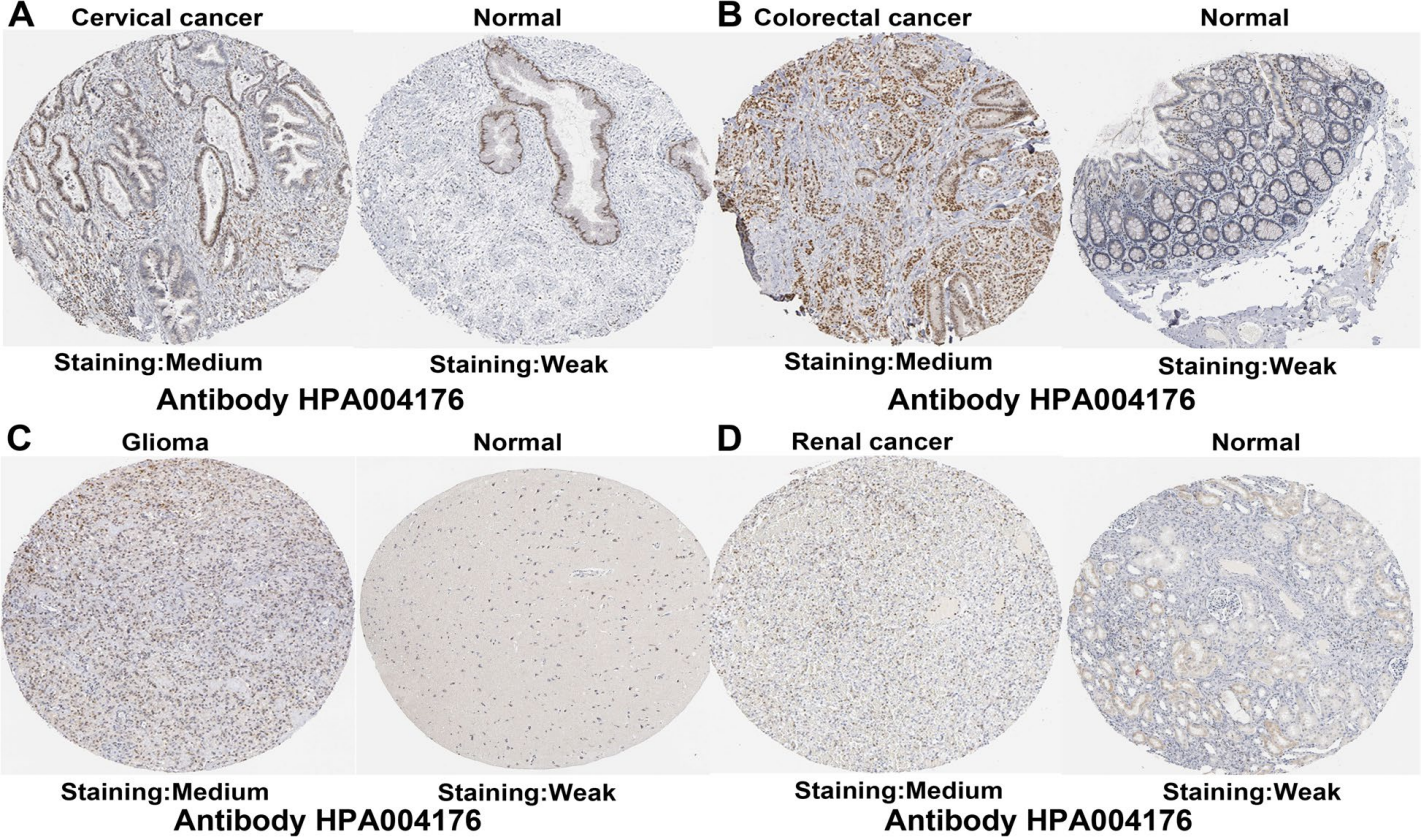
RUNX1 protein expression was detected by immunohistochemistry from the HPA database. **A**. The expression of RUNX1 protein in cervical cancer and normal sample; **B**. The expression of RUNX1 protein in colorectal cancer and normal sample; **C**. The expression of RUNX1 protein in glioma and normal sample; **D**. The expression of RUNX1 protein in renal cancer and normal sample

Based on the expression and prognosis results from the GEPIA database, RUNX1 may act as a potential prognostic biomarker for patients with cervical cancer, colorectal cancer, glioma, and renal cancer. Thus, we further verified RUNX1 protein expression in these cancers by immunohistochemistry, using the HPA database. The results showed that RUNX1 was expressed at a moderate level in tumor tissues, but very weak RUNX1 staining was detected in any normal tissue (Fig. 6A-D).

### *RUNX1* genetic alteration frequency analysis across human cancers

**Fig. 7:**
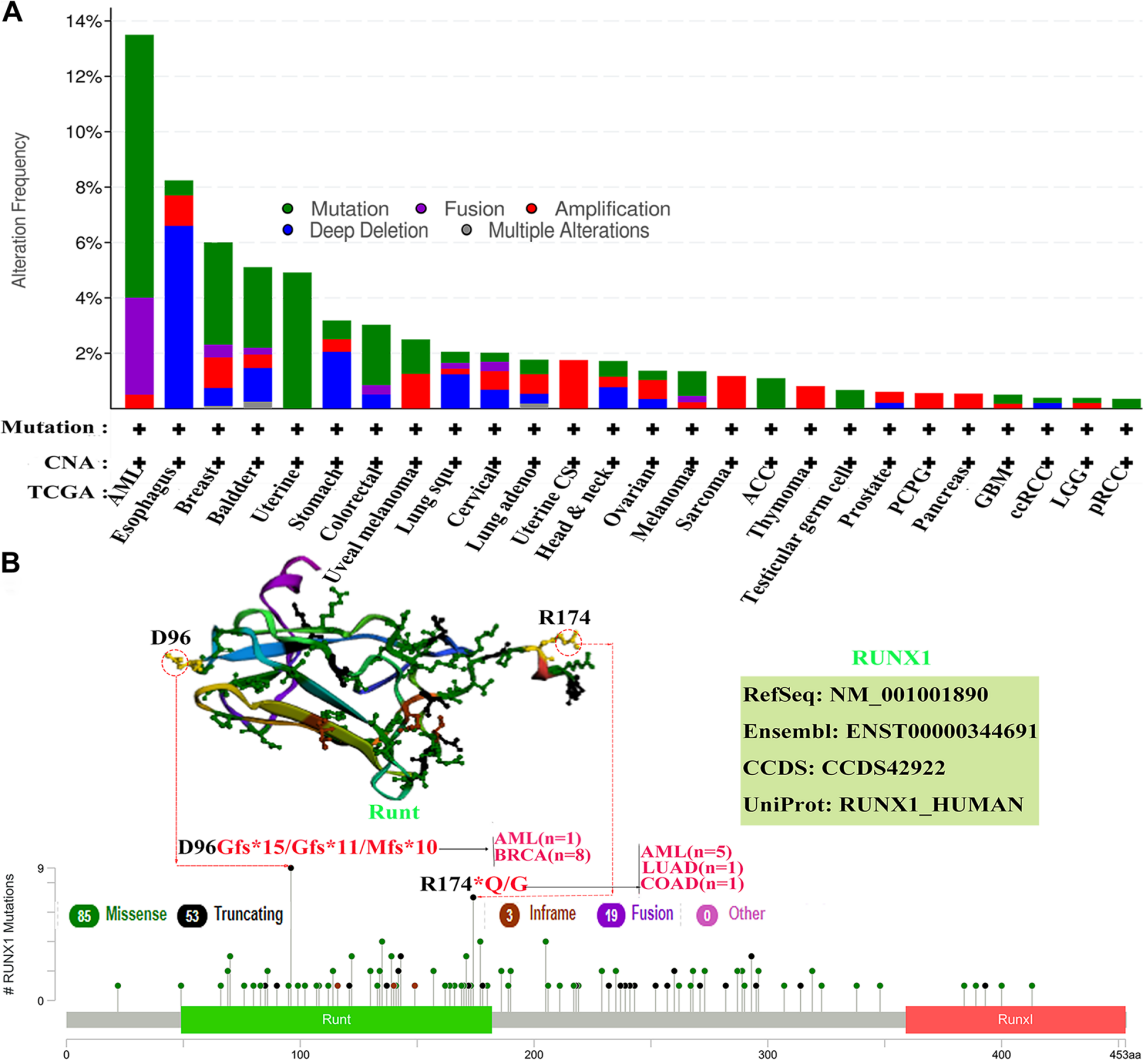
Mutation feature of *RUNX1* in different tumors (cBioPortal database). **A**. The alteration frequency of *RUNX1* with mutation type in human cancers based cBioPortal database; **B**.The mutation site with the highest alteration frequency (D96Gfs*15/Gfs*11/Mfs*10 and R174*Q/G) in the 3D structure of *RUNX1*

We searched the cBioPortal database to analyze the alteration frequency of the *RUNX1* gene in different cancers based on TCGA pan-cancer analyses. As shown in Fig. 7A, one or more alterations were detected in 33 types of human cancers, and 9.5% (19 cases) of patients with AML (200 cases) were observed to have mutations in *RUNX1,* representing the highest frequency among all patients with cancer. In addition, deep deletion of *RUNX1* occurred in 6.59% (12 cases) of patients with ESCA (182 cases), and mutations in *RUNX1* were the primary alteration type in 4.91% (26 cases) of patients with uterine cancer (529 cases). Subsequently, we queried the information of the genetic alterations of *RUNX1* in the pan-cancer analysis, including the type, site, and case number of each genetic alteration, in the cBioPortal database (Fig. 7B). The results indicated that *RUNX1* “Missense” mutation was the most common type of alteration among all. In addition, D96Gfs*15/Gfs*11/Mfs*10 alterations were observed in the Runt domain of RUNX1 in one case of AML and eight cases of BRCA. Whereas the R174*Q/G alteration in the Runt domain was observed in five cases of AML, one case of LUAD, and one case of COAD.

### Correlation analysis between RUNX1 expression and immune cells infiltration levels in diverse cancer types

**Fig. 8:**
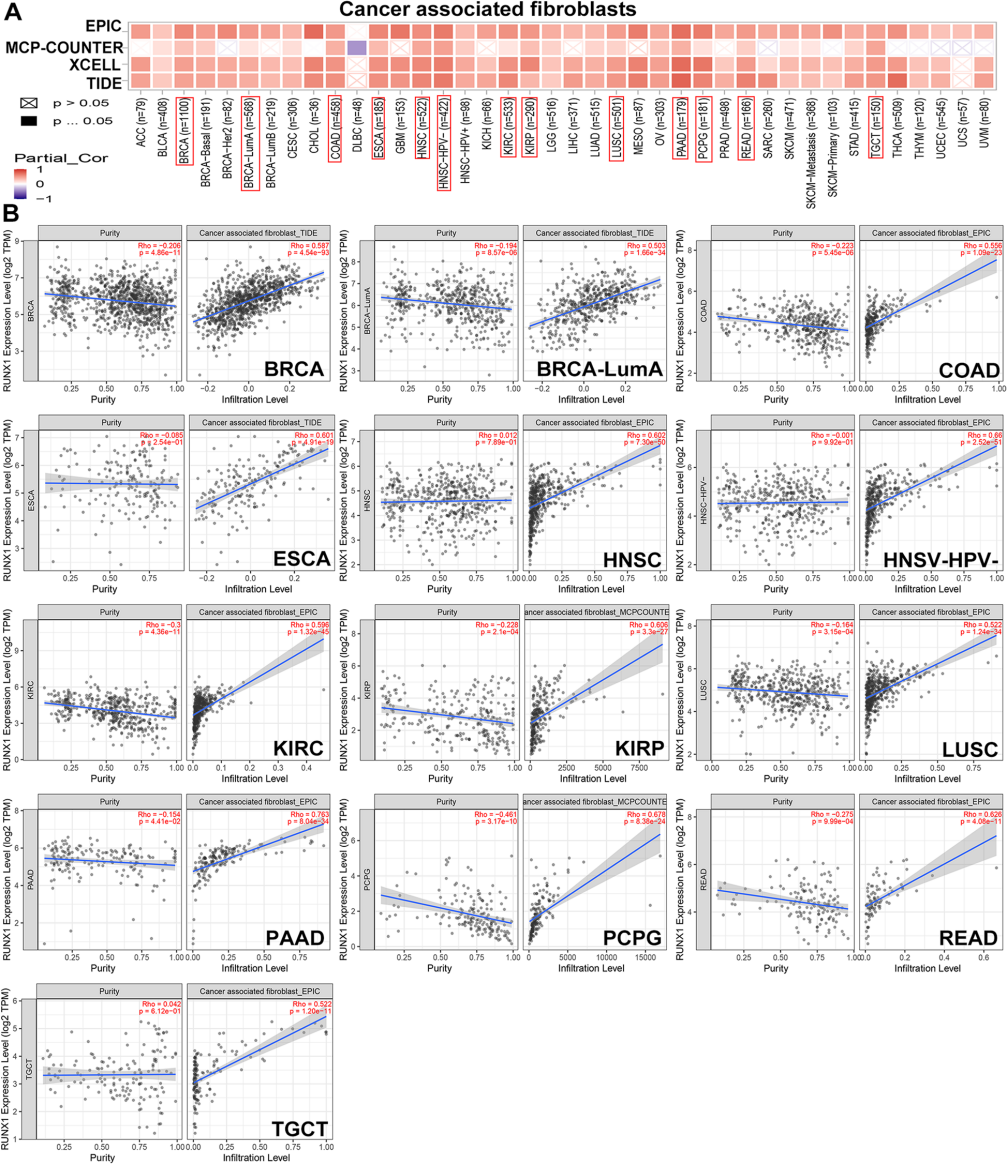
Correlation analysis between RUNX1 expression and immune infiltration of cancer-associated fibroblasts in TIMER database. **A**. The heat map indicated the correlation between RUNX1 expression and immune infiltration of cancer-associated fibroblasts; **B**. The significantly positive correlations between RUNX1 expression and immune infiltration of cancer-associated fibroblasts were observed in 13 kinds of tumors (Rho > 0.5)

In our study, we first analyzed the association between immune infiltration and RUNX1 expression in human cancers using TIMER 2.0. According to most algorithms, the infiltration level of CD8 + T cell was significantly negatively or positively associated with RUNX1 expression in all three tested tumors, including BRCA-Her2 (Rho = -0.379, *P* = 1.03e-03), lymphoid neoplasm diffuse large B-cell lymphoma (DLBC) (Rho = 0.532, *P* = 3.46e-4), and UVM (Rho = 0.665, *P* = 1.04e-10) (Fig. S3A-B). We also observed a significant positive correlation between CAFs infiltration and RUNX1 expression in 32 types of human cancer based on all algorithms (Fig. 8A-B).

**Fig. 9:**
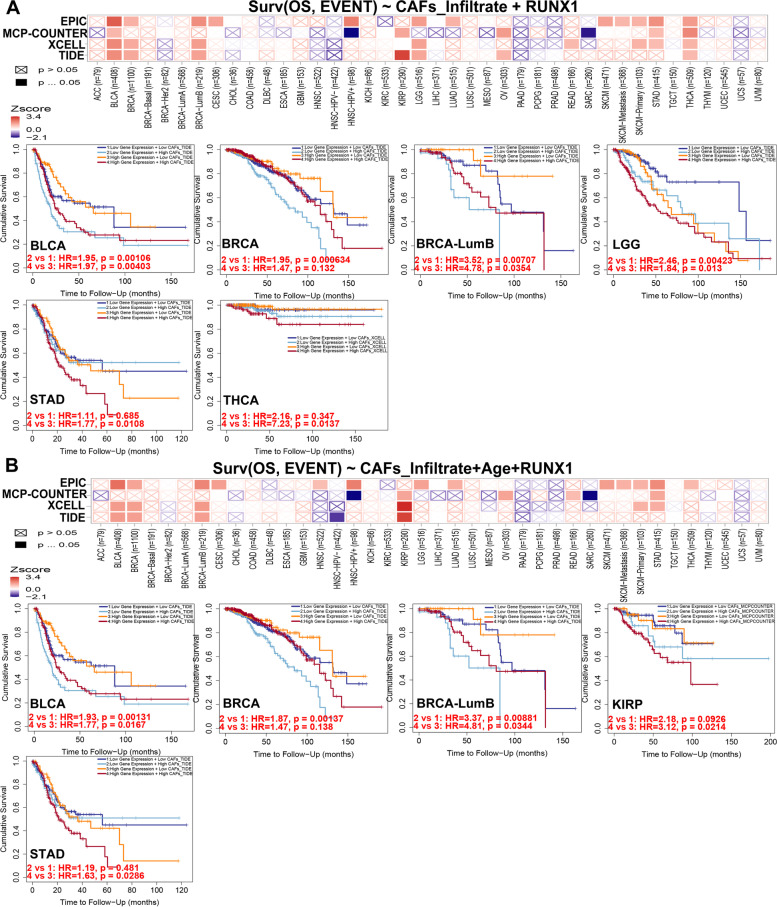
The prognostic signature built by the immune infiltration of cancer-associated fibroblasts and the expression of RUNX1 (TIMER database). **A**. The heat map and the survival curve indicated the correlation between RUNX1 experssion and infiltration of cancer-associated fibroblasts; **B**. The heat map and the survival curve indicated the correlation between the RUNX1 experssion and infiltration of cancer-associated fibroblasts by adjusting for confounders in the regression model

We used a Cox proportional hazard model to evaluate the effects of CAFs infiltration levels and RUNX1 expression on patient clinical outcomes in TIMER 2.0 database. CAFs infiltration was divided into low and high levels, and clinical factors and gene expression were selected as covariates. As shown in Fig. 9A, we revealed a prognostic correlation between RUNX1 expression and the infiltration of CAFs in various cancer types, according to most algorithms. RUNX1 may affect patient prognosis via immune infiltration of CAFs, including BLCA, BRCA, BRCA-LumB, LGG, STAD, and THCA. After adjusting for confounders (such as age, stage, sex, race, and tumor purity) in the regression model, we found a significant association between CAFs infiltration and RUNX1 expression in the prognosis of patients with cancer patients, such as BLCA, BRCA-LumB, CESC, LGG, READ, SARC (Table S3). The heat map and cumulative survival graphs for several cancers with statistical differences are displayed after adjusting for age factor (Fig. 9B). For example, as the age of cancer patients with high infiltration levels increases, the survival outcome is worse, including BLCA, BRCA, BRCA-LumB, KIRP, and STAD.

### RUNX1-related gene enrichment analysis across human cancers

**Fig. 10:**
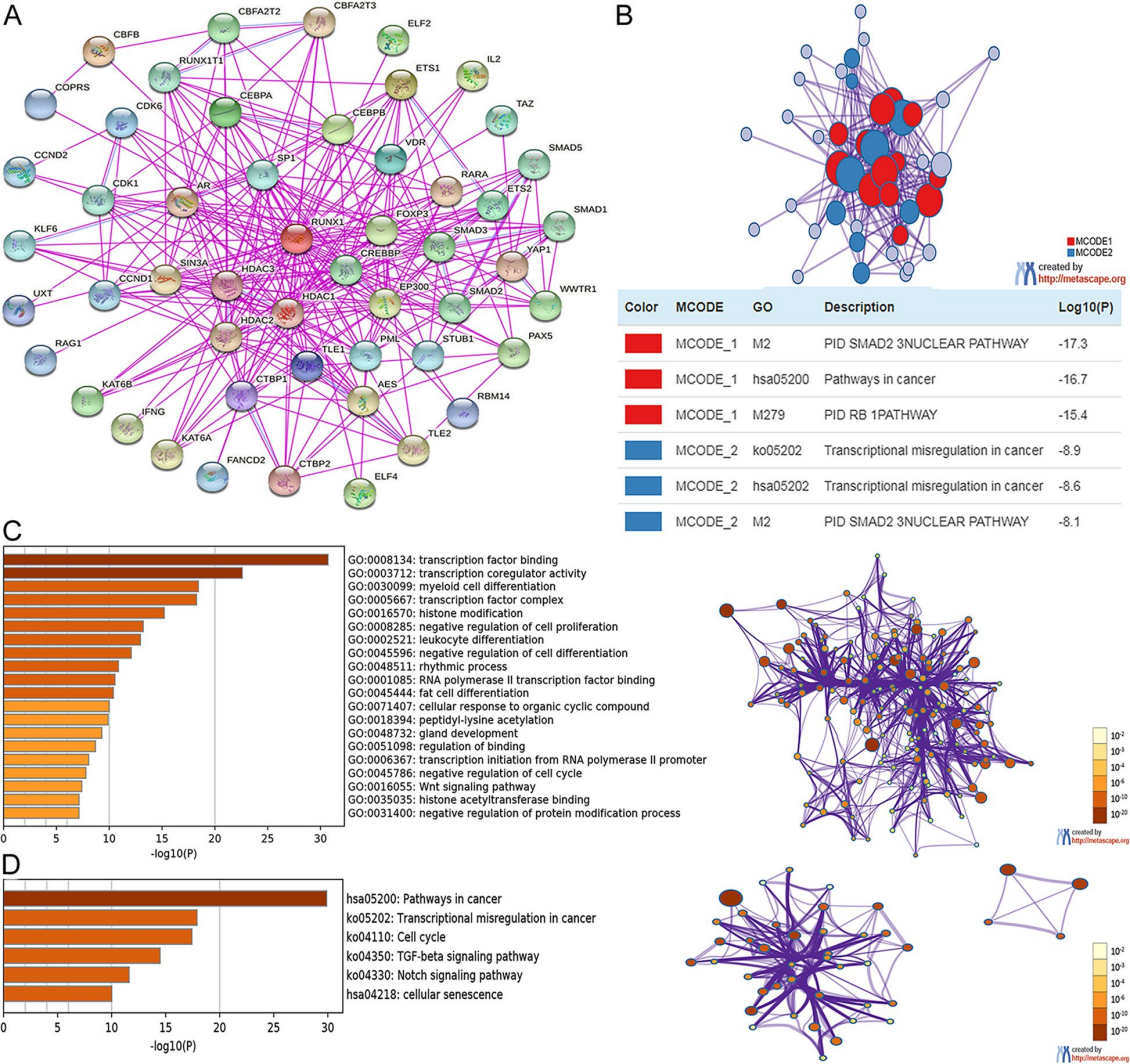
The enrichment analysis of RUNX1 and neighboring genes in human cancer. **A**. Protein–protein interaction network of RUNX1 (STRING database); **B**. PPI network and two most significant MCODE components form the PPI network; **C**. Heat map of Gene Ontology (GO) enriched terms colored by p-values; **D**. Heat map of Kyoto Encyclopedia of Genes and Genomes (KEGG) enriched terms colored by *p*-values

We conducted an enrichment analysis of *RUNX1* and its neighboring genes using STRING and Metascape databases. First, we identified neighboring genes interacting with *RUNX1* using the STRING website; the detailed results are shown in Fig. 10A. To better analyze the functional mechanism between RUNX1 expression and human cancers, we retrieved the Metascape database to construct a PPI enrichment analysis and obtained two of the most significant MCODE components from the analysis results (Fig. 10B).

Next, the potential functional mechanism of *RUNX1* and its neighboring genes was predicted using the Metascape database. We obtained the results of GO and KEGG analyses: GO analysis included biological process ( 15 items), molecular function (4 items), and cellular component (1 item) (Fig. 10C and Table 1). The top six KEGG pathways of RUNX1 and its neighboring genes are shown in Fig. 10D and Table 2.

**Table 1:** The Gene Ontology (GO) function enrichment analysis of RUNX1 and neighbor genes in pan-cancer (Metascape database)

Category (GO)	Term ID	Description	Count	Log10(*P*)
Molecular Functions	GO:0,008,134	transcription factor binding	25	-30.724
Molecular Functions	GO:0,003,712	transcription coregulator activity	20	-22.598
Biological Processe	GO:0,030,099	myeloid cell differentiation	16	-18.438
Cellular Components	GO:0,005,667	transcription factor complex	15	-18.268
Biological Processes	GO:0,016,570	histone modification	15	-15.205
Biological Processes	GO:0,008,285	negative regulation of cell proliferation	16	-13.220
Biological Processes	GO:0,002,521	leukocyte differentiation	14	-12.955
Biological Processes	GO:0,045,596	negative regulation of cell differentiation	15	-12.086
Biological Processes	GO:0,048,511	rhythmic process	10	-10.875
Molecular Functions	GO:0,001,085	RNA polymerase II transcription factor binding	8	-10.545
Biological Processes	GO:0,045,444	fat cell differentiation	9	-10.396
Biological Processes	GO:0,071,407	cellular response to organic cyclic compound	11	-9.988
Biological Processes	GO:0,018,394	peptidyl-lysine acetylation	8	-9.890
Biological Processes	GO:0,048,732	gland development	10	-9.297
Biological Processes	GO:0,051,098	regulation of binding	9	-8.692
Biological Processes	GO:0,006,367	transcription initiation from RNA polymerase II promoter	7	-8.054
Biological Processes	GO:0,045,786	negative regulation of cell cycle	10	-7.792
Biological Processes	GO:0,016,055	Wnt signaling pathway	9	-7.395
Molecular Functions	GO:0,035,035	histone acetyltransferase binding	4	-7.147
Biological Processes	GO:0,031,400	negative regulation of protein modification process	10	-7.145

**Table 2:** The Kyoto Encyclopedia of Genes and Genomes (KEGG) function enrichment analysis of RUNX1 and neighbor genes in pan-cancer (Metascape database)

Category	Term ID	Description	Count	Log10(*P*)
KEGG Pathway	hsa05200	Pathways in cancer	20	-29.897
KEGG Pathway	ko05202	Transcriptional misregulation in cancer	13	-17.899
KEGG Pathway	ko04110	Cell cycle	10	-17.428
KEGG Pathway	ko04350	TGF-beta signaling pathway	8	-14.481
KEGG Pathway	ko04330	Notch signaling pathway	6	-11.617
KEGG Pathway	hsa04218	cellular senescence	7	-10.015

## Discussion

As previously reported, RUNX1 expression plays a dual role in different human cancers. Increased RUNX1 expression is strongly associated with HNSC, predicting tumor recurrence and patient prognosis [26]. Several studies have demonstrated that RUNX1 expression is closely associated with the progression of solid tumors, such as ovarian cancer [9], glioblastoma [27], and renal cancer [28]. In contrast, RUNX1 overexpression has been linked to favorable outcomes and monitored prognosis during therapy in neuroblastoma [29]. RUNX1 expression can predict a better outcome for patients with prostate cancer, but plays a dual role in promoting or inhibiting the progression of this cancer [12]. In summary, RUNX1 expression may have positive or negative effects on the clinical outcomes of different cancers. In our study, the mRNA expression of RUNX1 was upregulated in almost all human tumors, according to the GenT2, TIMER, and GEPIA databases. However, RUNX1 expression also decreased in several cancer datasets from the GenT2 database. Thus, these findings urged us to further understand the expression status of RUNX1 in human cancers and its effect on tumor prognosis based on pan-cancer analysis.

Our analysis indicated that RUNX1 had a detrimental effect on four cancer types, including blood, brain, colorectal, and soft tissue cancers. In contrast, RUNX1 played a protective role in four other cancers. However, RUNX1 was found to have a negative overall effect on cancer survival according to the GEPIA database. RUNX1 expression was markedly linked to the prognosis of 11 cancer types (including OS and DFS) and had a detrimental effect on the corresponding tumors. However, RUNX1 had a protective role in both OS and DFS in patients with BRCA. Combining the results of the two databases, we found that the role of RUNX1 in ovarian cancer was detrimental. These differences may be associated with differences in data processing and molecular functions. The datasets in the PrognoScan database were obtained from GEO data, and the GEPIA datasets included TCGA and GTEx data. Considering the heterogeneity of multiple databases, we analyzed the potential prognostic value of RUNX1 based on TCGA data. Our results showed that RUNX1 overexpression may be a prognostic biomarker in several cancers, including CESC, COAD, GBM, and KIRC. Interestingly, RUNX1 expression in COAD was higher than in normal samples, suggesting a poor prognosis in these patients, which seems to be inconsistent with the role of tumor suppressors in gastrointestinal malignancies. This may be related to the genomic instability and diverse molecular functions of RUNX1 in gastrointestinal adenocarcinoma [30]. Additionally, overexpression of RUNX1 resulted in significantly poorer clinical survival in patients with renal cancer [28]. These results confirm that RUNX1 is a potential biomarker for these tumors.

RUNX1 is essential for the maturation of lymphocytes and megakaryocytes in adults [4]. Moreover, downregulated expression of RUNX1 has been linked to the regulation of myeloid-derived suppressor cells in lung cancer [31], whereas higher levels of RUNX1 can enhance the killing effect of natural killer cells in cervical cancer [32]. Furthermore, we observed that RUNX1 expression had negative or active effects on CD8 + T-cells infiltration in BRCA, DLBC, and UVM. Recent research has shown that RUNX1-related pathways may involve crosstalk between fibroblasts and tumor cells, and are closely related to the formation of CAFs [33]. We also observed a significant correlation between RUNX1 expression and CAFs infiltration in almost all tumors. RUNX1 may affect patient prognosis via immune infiltration of CAFs, which is closely related to tumorigenesis. Although causality cannot be established in the current study, these findings indicate that RUNX1 has a strong effect on the regulation of these immune-infiltrating cells and may affect the occurrence and progression of tumors.

Another major finding of our study was that RUNX1 expression correlated with diverse oncogenic signaling pathways in different cancers. RUNX1 is a crucial regulator of TGF-β signaling and plays an important role in several biological processes. Crosstalk between TGF-β signaling and other signaling pathways contributes to aberrant activation of cancer signaling, such as those of the Wnt/β-catenin and Notch signaling pathways [34]. Moreover, the transcription factor RUNX1 enhances the Wnt/β-catenin signaling pathway, thereby promoting tumor progression and metastasis in colorectal cancer [35]. Therefore, it is reasonable to speculate that RUNX1 expression may influence patient prognosis by activating oncogenic signaling pathways in human cancers.

In summary, we systematically analyzed the effect of RUNX1 expression on the prognosis of several human malignancies. Our results suggest that RUNX1 expression is dysregulated in almost all tumors and may have positive or negative effects on the clinical outcomes of patients with cancer. Additionally, an increased level of RUNX1 is associated with the immune infiltrate levels of CAFs and the activation of oncogenic signaling pathways in human cancers, which could be the potential mechanism by which RUNX1 affects patient outcomes. However, further verified experiments have not been performed in human cancers, limiting the analysis of the data. Nevertheless, the findings of our study contribute to a comprehensive understanding of the underlying mechanisms of RUNX1 in human tumors and provide support for subsequent experimental studies.

## Conclusions

We found that RUNX1 expression correlates with patient outcomes and immune infiltrate levels of CAFs in multiple tumors. These findings suggest that RUNX1 can function as a potential prognostic biomarker and reflect the levels of immune infiltrates of CAFs in human cancers.

## Supplementary Information

**Additional file 1:** **Fig. S1.** Pan-canceranalysis of RUNX1 mRNA expression level across cancers in the GEPIA database. **Fig. S2. **Pan-canceranalysis of RUNX1 protein expression level across cancers in the Human Protein Atlas. A. Protein expression of RUNX1 in normal tissuesB. Protein expression of RUNX1 in cancer tissues. **Fig. S3.** Correlationanalysis between RUNX1 expression and immune infiltration of CD8+ T-cells inTIME database. A. Thecorrelation between RUNX1expression and immune infiltration of CD8+ T-cells in pan-cancer. B.The immune infiltration of CD8+ T-cells and RUNX1 expression was a significant positive correlation inBRCA-Her2, DLBC, and UVM. **Table S1.** Pan-cancer survivalanalyses of RUNX1 in the 13 types of cancer from PrognoScan database. **Table S2. **Pan-cancersurvival analyses of RUNX1 in the 33types of cancer from GEPIA database. **Table S3. **The Coxregression analysis for evaluating the prognostic value of the risk score based on TIMER 2.0 database.

## Data Availability

The data of the current study is available from the following open public databases: TCGA (https://cancergenome.nih.gov), GTEx (https://gtexportal.org/home/datasets), and Metascape (https://metascape.org/gp/index.html) as is described above. Other data will be obtained from the corresponding authors upon reasonable request.

## Abbreviations

BLCA: Bladder urothelial carcinoma
BRCA: Breast invasive carcinoma
CAFs: Cancer-associated fibroblasts
CBF-β: Core binding factor-beta
CC: Cellular component
CESC: Cervical squamous cell carcinoma and endocervical adenocarcinoma
CHOL: Cholangiocarcinoma
COAD: Colon adenocarcinoma
DFS: Disease-free survival
DLBC: Lymphoid neoplasm diffuse large B-cell lymphoma
ESCA: Esophageal carcinoma
GBM: Glioblastoma multiforme
GEO: Gene Expression Omnibus
GEPIA: Gene Expression Profiling Interactive Analysis
GO: Gene Ontology
GTEx: Genotype-Tissue Expression
HNSC: Head and neck squamous cell carcinoma
HPA: Human Protein Atlas
KEGG: Kyoto Encyclopedia of Genes and Genomes
KIRC: Kidney renal clear cell carcinoma
KIRP: Kidney renal papillary cell carcinoma
LAML: Acute myeloid leukemia
LIHC: Liver hepatocellular carcinoma
LGG: Brain lower-grade glioma
LUAD: Lung adenocarcinoma
LUSC: Lung squamous cell carcinoma
MESO: Mesothelioma
MF: Molecular function
OS: Overall survival
RUNX1: Runt-related transcription factor 1
RUNXs: Runt-related transcription factors
TCGA: The Cancer Genome Atlas
TIMER: Tumor Immune Estimation Resource
OV: Ovarian cancer
PAAD: Pancreatic adenocarcinoma
PCPG: Pheochromocytoma and paraganglioma
PRAD: Prostate adenocarcinoma
READ: Rectum adenocarcinoma
SARC: Sarcoma
SCKM: Skin cutaneous melanoma
STAD: Stomach adenocarcinoma
TGCT: Testicular germ cell tumors
THCA: Thyroid carcinoma
THYM: Thymoma
UCEC: Uterine corpus endometrial carcinoma
UCS: Uterine carcinosarcoma
UVM: Uveal melanoma
