# Supplementary material for: RUNX1 is a promising prognostic biomarker and related to immune infiltrates of cancer-associated fibroblasts in human cancers
Source: BMC Cancer. 2022 May 9;22:523. doi: 10.1186/s12885-022-09632-y (PMC9088136; doi:10.1186/s12885-022-09632-y)
Supplement: Supplementary file 1 — Additional file 1: Fig. S1. Pan-canceranalysis of RUNX1 mRNA expression level across cancers in the GEPIA database. Fig. S2. Pan-canceranalysis of RUNX1 protein expression level across cancers in the Human Protein Atlas. A. Protein expression of RUNX1 in normal tissuesB. Protein expression of RUNX1 in cancer tissues. Fig. S3. Correlationanalysis between RUNX1 expression and immune infiltration of CD8+ T-cells inTIME database. A. Thecorrelation between RUNX1expression and immune infiltration of CD8+ T-cells in pan-cancer. B.The immune infiltration of CD8+ T-cells and RUNX1 expression was a significant positive correlation inBRCA-Her2, DLBC, and UVM. Table S1. Pan-cancer survivalanalyses of RUNX1 in the 13 types of cancer from PrognoScan database. Table S2. Pan-cancersurvival analyses of RUNX1 in the 33types of cancer from GEPIA database. Table S3. The Coxregression analysis for evaluating the prognostic value of the risk score based on TIMER 2.0 database. [file 12885_2022_9632_MOESM1_ESM.docx]

**Additional fle 1: Fig. S1.** Pan-cancer analysis of RUNX1 mRNA expression level across cancers in the GEPIA database.

**Additional fle 2: Fig. S2.** Pan-cancer analysis of RUNX1 protein expression level across cancers in the Human Protein Atlas. A. Protein expression of RUNX1 in normal tissues. B. Protein expression of RUNX1 in cancer tissues.

**Additional fle 3: Fig. S3.** Correlation analysis between RUNX1 expression and immune infiltration of CD8+ T-cells in TIME database. A. The correlation between RUNX1expression and immune infiltration of CD8+ T-cells in pan-cancer. B.The immune infiltration of CD8+ T-cells and RUNX1 expression was a significant positive correlation in BRCA-Her2, DLBC, and UVM.

**Additional fle 4: Table S1.** Pan-cancer survival analyses of RUNX1 in the 13 types of cancer from PrognoScan database.

**Additional fle 5: Table S2.** Table S2. Pan-cancer survival analyses of RUNX1 in the 33 types of cancer from GEPIA database.

**Additional fle 6: Table S3.** The Cox regression analysis for evaluating the prognostic value of the risk score based on TIMER 2.0 database.
